# Optimization of process parameters in micro-scale pneumatic aerosol jet printing for high-yield precise electrodes

**DOI:** 10.1038/s41598-023-47544-4

**Published:** 2023-12-02

**Authors:** Hakyung Jeong, Jae Hak Lee, Seungman Kim, Seongheum Han, Hyunkyu Moon, Jun-Yeob Song, Ah-Young Park

**Affiliations:** Department of Ultra-Precision Machines and Systems, Korea Institute of Machinery and Materials (KIMM), Daejeon, 34103 Republic of Korea

**Keywords:** Mechanical engineering, Electrical and electronic engineering

## Abstract

Aerosol jet printing (AJP) is a new non-contact direct writing technique designed to achieve precise and intricate patterns on various substrates. Specifically, the pneumatic AJP process breaks down the ink into fine particles, significantly reducing the risk of nozzle clogging and rendering it highly advantageous for industrial applications. This paper focuses on the optimization of the line electrode formation process using soluble silver clusters as the conductive ink, along with the aerosol formation procedure. The main parameters of the AJP process, namely sheath flow rate, atomizer flow rate, and dispensing speed, were identified and examined for their influence on line width and resistivity. Through this analysis, an operability window, including optimized conditions for printing high-quality lines using the AJP process, was established, along with a regression equation enabling the statistical estimation of line width. In summary, the outcomes of this investigation underscore the feasibility of an integrated printing system capable of precision control over line width, achieved through the optimization of AJP process parameters. Furthermore, it was established that pneumatic AJP offers robust process stability. The practical applicability of the proposed optimization techniques was assessed, highlighting their potential utilization in electrode formation processes within the electronic and display industry.

## Introduction

In recent years, extensive research has been undertaken to explore the potential of the printing process for fabricating cost-effective, large-area electronic circuits and devices on flexible electronic substrates^1^. Various methods have been explored, including screen printing^2,3^, inkjet printing^4–6^, roll-to-roll printing^7–9^, gravure printing^10,11^, and aerosol printing^12–14^. Given the intricate requirements of the printing process for electronic devices, such as organic light-emitting diodes and thin-film transistors which often involve multi-layered structures, precise control over layer sizes and placements is essential^15–17^. Moreover, as electronic devices include diverse materials, the printing process must accommodate inks with varying properties^18^. Among these techniques, the Aerosol Jet Printing (AJP) method has been focused as the most efficient production applied to a roll-to-roll process for large-area electronic circuits and devices. In addition, the AJP technology extends to its ability to generate conductors^12,19,20^, semiconductors^21–23^, and insulators^24–26^, earning recognition as a valuable tool for various applications involving transistors, strain gauges, interconnects, electrode arrays, and more.

Aerosol printing stands as a relatively new technique within the domain of printed electronic device manufacturing^27,28^. This method involves the aerosolization of functional ink, propelled by gas streams. Aerosolization is achieved through both pneumatic and ultrasonic means^29^. The aerosol stream is directed towards the print head, precisely focused through coaxial sheath gas flow. This process yields a dense deposit of a pre-defined size based on the nozzle dimensions.

This paper investigates the process of generating conductive lines through aerosol printing. The fabrication of conductive lines demands adequate thickness for narrow widths and high current-carrying capacity for densely packed circuits. Hence, the aim of printing conductive networks lies in achieving lines with both high resolution (minimal line width) and a substantial aspect ratio (thickness-to-width ratio)^30,31^. However, attaining these objectives often poses challenges in maintaining a high printing speed. In the context of aerosol printing, producing a thick line requires multiple processes, ultimately increasing the width of the printed line^32,33^.

While most previous research has highlighted the numerical investigation^34^ and application of aerosol printing for new materials or electronic devices, fewer studies have explored analyzed process conditions that influence print quality at the industrial standard. Firstly, concerning materials, the addition of a surfactant to silver ink increased its electrical property beyond the limitation of the aerosol process^35^. Other cases focus on the aspect ratio of the line pattern achieved by heating the substrate or controlling the operating parameters of aerosol jet printing^36^. However, it is challenging to employ this approach in multi-layer electronic devices due to the high thickness of electrodes, which can render them susceptible to mechanical deformation. Furthermore, the fabrication of electronic devices actively employs aerosol jet technology to enhance performance and simplify processes. A study similar to this paper, involving silver printing through pneumatic aerosol methods, revealed that line width was influenced by three adjustable flow rates and the stage speed of the machine, and the quality of printed line edges changed by manipulating three gas flow rates using pneumatic aerosol equipment^12,32,37^. However, the focus on thickness control primarily revolved around the impact of the deposited layers. Beyond the scope of these studies, the ultimate goal of production equipment is the development of practical processes that can be utilized in the industry. Therefore, the applicability of the pneumatic aerosol jet printing process should be statistically validated.

This study aims to comprehensively investigate the interplay between process parameters, such as sheath flow rate, atomizer flow rate, and dispensing speed. Their impact on the morphology and electrical characteristics of lines generated through pneumatic aerosol equipment employing inorganic materials, specifically silver clusters. The optimized conditions yield high-quality line patterns that guarantee resistivity and can be estimated using a regression equation calculated through statistical analysis.

## Results

### Fundamentals of aerodynamics in pneumatic aerosol jetting

**Figure 1:**
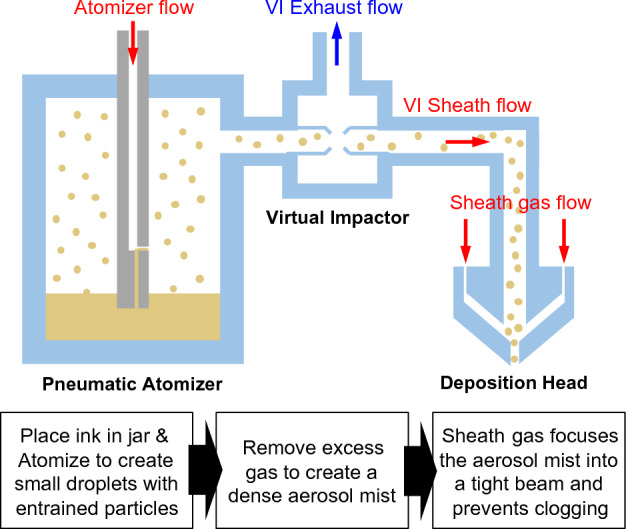
A schematic of the AJ system printing mechanism with aerodynamic flows.

The AJ process employs aerodynamic focusing to achieve high-resolution deposition of colloidal suspensions and/or chemical precursor solutions. An aerosol stream containing the deposition material is meticulously directed, deposited, and structured onto either a planar or a 3D substrate^38–40^. This fundamental system comprises two essential components, as demonstrated in Fig. 1: a module responsible for atomizing liquid raw materials (mist generation), and a second module dedicated to focusing the aerosol and depositing the droplets (in-flight processing). The mist generation is accomplished through the utilization of an ultrasonic or pneumatic atomizer. Subsequently, the aerosol stream is precisely concentrated utilizing a flow deposition head, which establishes a circular, coaxial flow between the aerosol stream and a sheath gas stream. This co-axial flow exits the print head via a nozzle directed towards the substrate. The AJ print head exhibits the capability to concentrate an aerosol stream down to a tenth of the size of the nozzle orifice. The system’s distinctive ability to print on non-planar surfaces makes it an optimal solution for producing complicated 3D shape of sensors and displays. This feat is made possible by the relatively substantial stand-off distance (1–5 mm) of the deposition head above the substrate, coupled with the extended focal length of the material beam emerging from the nozzle. Notably, there exists no physical contact between any part of the tool (except for the deposition stream) and the substrate, thereby facilitating the achievement of seamless, conformal writing.

The final flow rate at the deposition head is calculated by Eqs. (1), (2) as below^41^:

1 $$Q_{Push} = Q_{Atomizer} + Q_{VI Sheath} - Q_{VI Exhaust}$$

2 $$Q_{Total\;Nozzle} = Q_{Push} + Q_{Sheath}$$

where Q is the flow rate in sccm and Q_Push_ is the flow rate of the aerosol mist to the print head. The AJ printing system consists of three steps as shown in Fig. 1. The first step involves the formation of aerosol in the pneumatic atomizer and its flow through the virtual impactor. The pneumatic atomizer exploits high-velocity gas stream to generate a liquid stream into a droplet size of 1–5 μm. The atomizer connected with add-back module introduces solvent to the atomizer to maintain desired ink viscosity for consistent output. Solvents with low to medium vapor pressure are suitable, but high vapor pressure solvents can lead to excessive drying and powdery deposits. Atomizer flow rate impacts atomization rate, adjustable from 0 to 2000 sccm. Typical rates are 800–1500 sccm, but some materials may need higher rates for acceptable atomization. The optimal atomizer flow rate depends on material viscosity.

The second step is a process in which the aerosol stream from the virtual impactor (VI) is filtered to flow smoothly, and excess gas is removed to increase the density of the material. In this step, a solvent add-back module is used, which adds solvent to sheath gas, saturating atomized aerosol to prevent VI blockage due to drying. The VI sheath flow is set to be the same as the atomizer flow rate. As shown in Eq. (1), the determined flow rate at this point becomes the push flow rate to the deposition head.

The third step is the process of printing through the nozzle in the deposition head. Sheath gas flow focuses aerosol mist, defining printed line width. Low flow can reduce product quality; high flow can disrupt printing. Sheath process parameters are often communicated using sheath-to-push ratio, acceptable range depending on nozzle size. The boost and divert flow rates in the deposition head, responsible for redirecting the aerosol stream, will both be set to match twice the selected push flow rate. During process development, the total nozzle flow rate is determined by combining the push and sheath flow rates as shown in Eq. (2). Dispensing height refers to the measurement from the surface of substrate to the nozzle’s tip. Various nozzles necessitate varying dispense heights, with a common guideline being 10 times the nozzle diameter.

The pneumatic aerosol printing is influenced by controllable factors: atomizer flow rate, VI sheath flow rate, sheath flow rate, nozzle diameter, dispensing height, dispensing speed, and stage/head temperature. These variables can be categorized into primary variables (atomizer flow rate, VI sheath flow rate, sheath flow rate, dispensing speed)^12,27,32^ affecting printability and additional variables (nozzle diameter, dispensing height, stage/head temperature)^36,42–44^. The primary variables are universal and applicable to a wide range of aerosol jet printing systems and additional variables can vary significantly depending on the specific application, materials, and substrates being used. This industrialization strategy enables applicability to all of aerosol jetting equipment. Among the previously defined primary variables, three critical independent factors were selected for conducting factor analysis experiments: (1) sheath flow rate, (2) atomizer flow rate, and (3) dispensing speed. Considering the process parameters, the dependent variables also include the silver ink with suitable properties, along with the dispensing height and stage temperature^28,41^.

### Influence of critical process parameters in printing quality

**Figure 2:**
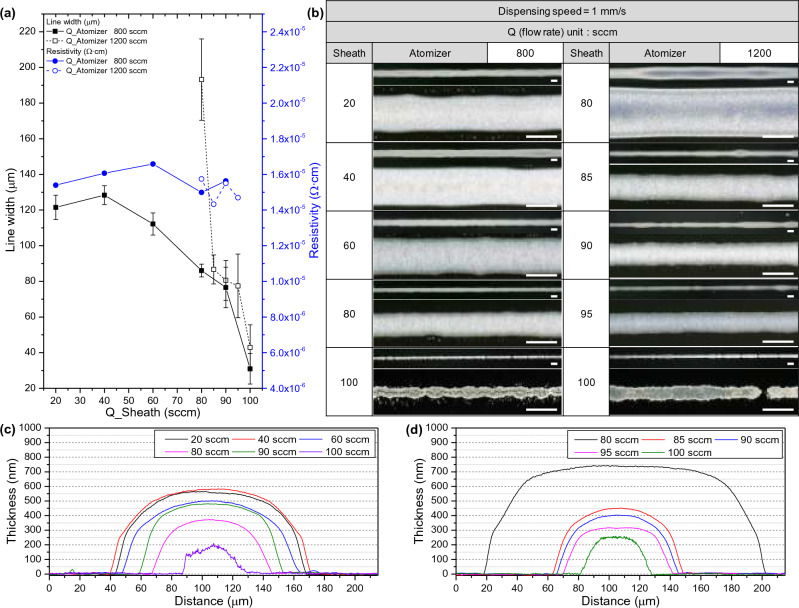
Dependence of the sheath flow rate in the AJ system printing: (**a**) Line width and resistivity and (**b**) optical microscopy (scale bar = 100 μm) results under the same dispensing speed and atomizer flow rate. Comparison of thickness according to sheath flow rate using 3D profiler at atomizer flow rate of (**c**) 800, (**d**) 1200 sccm.

The aerosol stream is conveyed through a polypropylene tube from the VI to the ceramic deposition nozzle. The ceramic nozzle used has an internal diameter of 300 μm. The deposition head contains nitrogen sheath gas, which aerodynamically focuses or pinches the aerosol stream as it enters the ceramic nozzle. The flow rate of the independent sheath gas can be controlled through a mass flow controller (MFC). The sheath gas is a guiding gas through the nozzle to stabilize the jetting of the ink. By adjusting the amount of sheath gas, the line width and thickness of the pattern can be controlled. Figure 2 illustrates the printed line electrodes based on various sheath flow rates at two different atomizer flow rates of 800 and 1200 sccm. The viscosity of the ink used in experiments is 10 cPs, with further details elaborated in the Methods section below. Due to the variation in the amount of aerosol ejected depending on the atomizer flow rate, it is necessary to set an appropriate sheath flow rate. As the sheath flow rate increases, the ink ejection relatively decreases, leading to a reduction in line width shown in Fig. 2a,b. However, when the sheath flow rate exceeds an appropriate level, the quality of the lines diminishes. The sheath flow rate operates much like when we water a garden using a hose. Just as we gently squeeze the hose’s end to propel the water farther and create a controlled stream, the sheath flow rate concentrates the aerosol to produce fine patterns. However, akin to turning the hose’s nozzle too high, setting the sheath flow rate excessively can have adverse effects, jeopardizing process stability and even leading to short circuits. When the sheath flow rate is 100 sccm, an increase in the atomizer flow rate results in line pattern shorts, indicating that optimizing sheath flow rate is essential for achieving high quality line patterns. The resistivity was measured around 1.6 × 10^−6^ Ω·cm^2^ in well-printed areas, regardless of the process conditions. When the sheath flow rate exceeded 100 sccm, the quality of the lines reduced to dots that measurable resistance values could not be obtained. Sheath gas is important for focusing efficiency as it concurrently induces the drying of droplets around the periphery of the aerosol by aerodynamics^28^. An incomplete aerosol stream results in short circuit and a rapid increase in resistivity as shown in Fig. 2a,b. Figure 2c,d demonstrated that, at a constant atomizer flow rate of 800 and 1200 sccm, an increase in sheath flow rate led to a decrease in line width and subsequently thickness.

**Figure 3:**
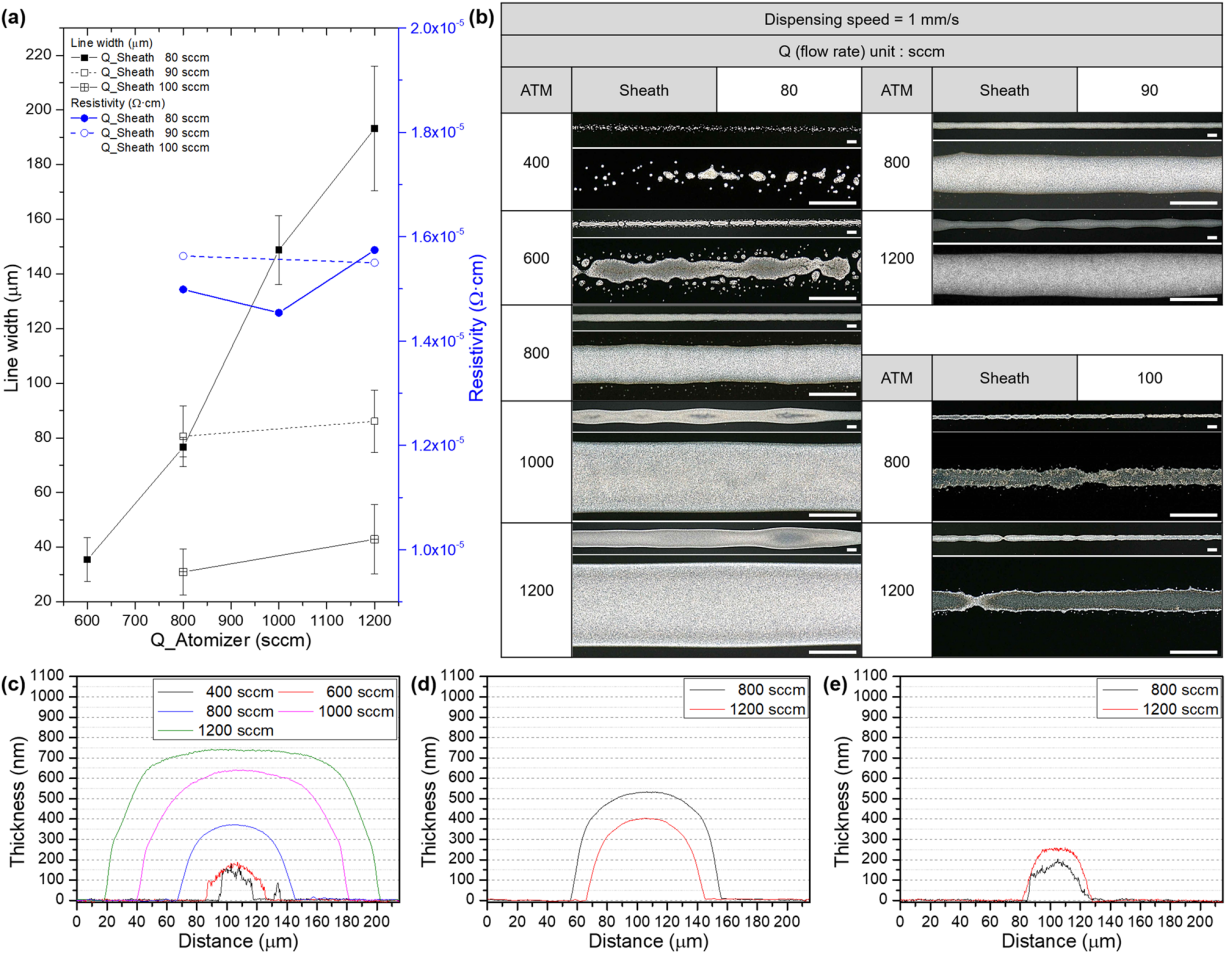
Dependence of the atomizer flow rate in the AJ system printing: (**a**) Line width and resistivity and (**b**) optical microscopy (scale bar = 100 μm) results under the same dispensing speed and sheath flow rate. Comparison of thickness according to atomizer flow rate using 3D profiler at sheath flow rate of (**c**) 80, (**d**) 90, (**e**) 100 sccm.

The atomizer process occurs within the atomization jar to generate an aerosol stream from the ink. Incoming nitrogen gas carries the mist of fine droplets from the exhaust port of the atomization jar after atomizing the ink. Conversely, larger droplets, due to their significant inertial momentum, remain unaffected by the nitrogen and stay in place. In the case of pneumatic atomization, the aerosol stream is formed by the nitrogen gas flowing vertically through the ink, regulated by the atomizer (ATM) flow rate, allowing for precise control over the amount of atomized ink. The atomized stream contains ink droplets, some of which are large enough to potentially clog the nozzle. The virtual impactor sheath (VIS) is positioned behind the atomizer, as depicted in Fig. 1. The VIS includes multiple vacuum inlets that can filter the aerosol stream. Ink droplets with lower inertial momentum, which tend to form satellites during printing, are carried along by the nitrogen gas and subsequently expelled through a filter. Larger droplets are filtered out through the vacuum inlets, allowing only appropriately sized droplets to pass through and be printed via the nozzle. As previously mentioned, the VIS flow rate is set to match the ATM. Figure 3 illustrates the printing trend of line patterns based on the ATM. Results of line width in Fig. 3a,b demonstrate effects of the ATM on the quality of printed lines with three different sheath flow rates. It shows that an increase in the ATM leads to greater atomized ink volume, resulting in increased ejection rate and consequently wider pattern width. When the flow rate is insufficient, the distilled water through the solvent add-back module may generate voids or shorts in the pattern. Furthermore, higher sheath flow rates also elevate the chance of instability and shorts in pattern printing as the atomizer flow rate increases. When aerosol is jetted at high sheath gas flow rates causes the aerosol to dry, unstable flow is formed^28^. This results in the inability to measure resistivity. Therefore, optimizing appropriate ranges of sheath and atomizer flow rates is essential depending on the ink properties. Resistivity maintains levels around 1.5 × 10^−6^ Ω cm^2^ when printing proceeds readily. The resistivity is directly influenced by the ATM flow rate, as evidenced in Fig. 3a. When the ATM flow rate reduces below the minimum rate required for printing, the absolute volume of the generated aerosol becomes insufficient, leading to short circuit of electrodes. Thickness results in Fig. 3c–e also show a proportional increase in line width and thickness as the atomizer flow rate increases.

**Figure 4:**
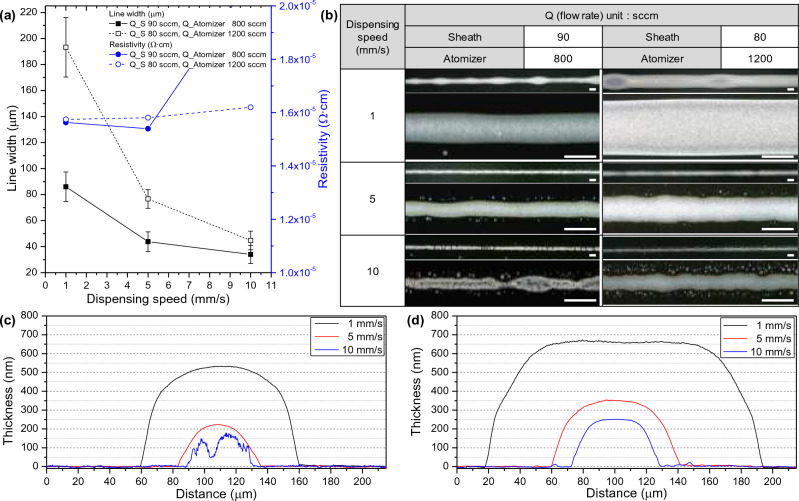
Dependence of the dispensing speed in the AJ system printing: (**a**) Line width and resistivity and (**b**) optical microscopy (scale bar = 100 μm) results under the same sheath and atomizer flow rate. Comparison of thickness according to atomizer flow rate using 3D profiler at sheath, atomizer flow rate of (**c**) 90, 800, and (**d**) 80, 1200 sccm.

Dispensing speed signifies the velocity of the print head comprising the nozzle. Figure 4 represents the influence of dispensing speed at various sheath and atomizer flow rates. Dispensing speeds of 1, 5, and 10 mm/s were compared under two conditions of sheath and atomizer flow rates selected from previous experiments. In Fig. 4a,b, increasing the dispensing speed results in a reduced ink deposition per unit area of the substrate, leading to the formation of thinner line widths with an increased presence of satellites. From an aerodynamic perspective, particularly in the ink injection system, the eddy shedding, which is the circulation of ink around the substrate, is influenced by the printing frequency and speed^45^. This is the fundamental cause of satellite formation, and when a large number of satellites are produced, they coalesce to make the line width broader and well-defined, whereas a smaller number of satellites result in a satellite state. The quantity of satellites is determined by the dispensing speed, and increasing the focusing efficiency reduces this phenomenon^28^. Consequently, within the scope of this experiment, the ink used appears to be suitable within the range of 1–5 mm/s. Resistivity values also exhibit consistent measurements across all conditions, around 1.6 × 10^−6^ Ω cm^2^, suggesting successful printing, with the exception of conditions where appears to be an insufficient quantity of aerosol at high dispensing speed. Figure 4c,d show a tendency of decreased line width and thickness as the speed increases. However, due to the viscosity of the ink, the thickness does not exceed 700 nm and tends to spread in the direction of line width. Dispensing speed serves as a factor that allows for easy control of line width while maintaining high printing quality. By considering the desired line width and quality, an appropriate dispensing speed can be determined.

**Figure 5:**
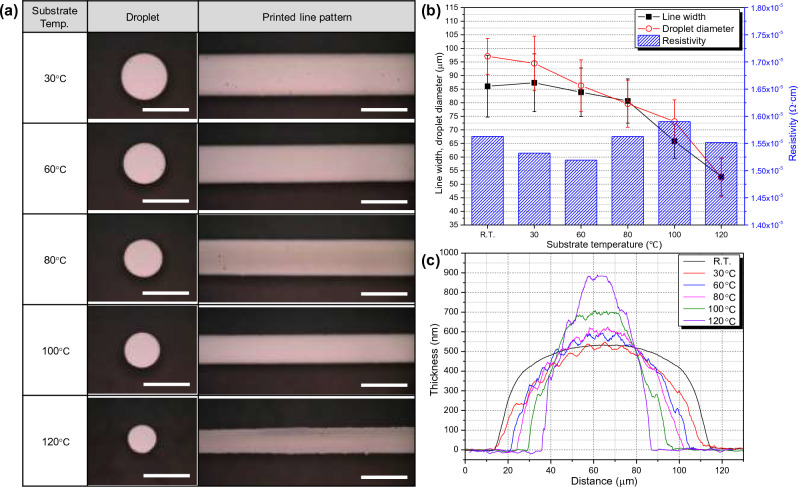
Influence of the substrate temperature in the AJ system printing for five temperatures (30, 60, 80, 100, 120°C): (**a**) The morphology of droplet and printed line patterns by optical microscopy (scale bar = 100 μm). Comparison of (**b**) line width, droplet diameter, resistivity, and (**c**) thickness according to substrate temperature using 3D profiler.

Furthermore, the primary parameters defined previously, additional parameters were existed. Among these, substrate temperature influences the drying process after printing, potentially impacting print uniformity, morphology, and resolution^28^. The print morphology corresponding to substrate temperature is described in Fig. 5. The substrate temperatures were compared under five conditions: 30, 60, 80, 100 and 120°C. In this experiment, glass substrates were placed on a stage set to a specific temperature, allowing for temperature stabilization before printing. AJP conditions were set as atomizer, sheath flow rate, and dispensing speed of 800, 90 sccm and 1 mm/s, respectively. Subsequently, curing was performed for 5 min on a 150°C hotplate. The results from the optical microscope images in Fig. 5a indicated a general tendency of decreased line width and droplet diameter with increasing substrate temperature. The narrower line width caused by higher substrate temperatures can be attributed to the decrease in solvent evaporation rate and the spread time of printed ink^37^. When considering the averages and variances of droplet diameter and line width in Fig. 5b, the trends align consistently. The resistivity was observed similar values around 1.55 × 10^−6^ Ω cm^2^ because of equal curing process. Figure 5c illustrates the results of the thickness and width of the printed line pattern. Since the quantity of ink printed under all conditions was the same, a decrease in width led to an increase in thickness, keeping the integral area (volume) of the line pattern constant. Due to the characteristics of the ink used in this study, insignificant difference is observed in line width at temperatures below 60°C, but a significant decline is noticed at temperatures above 80°C. Therefore, the extent to which substrate temperature influences the outcome could determine by the characteristics of the ink.

**Figure 6:**
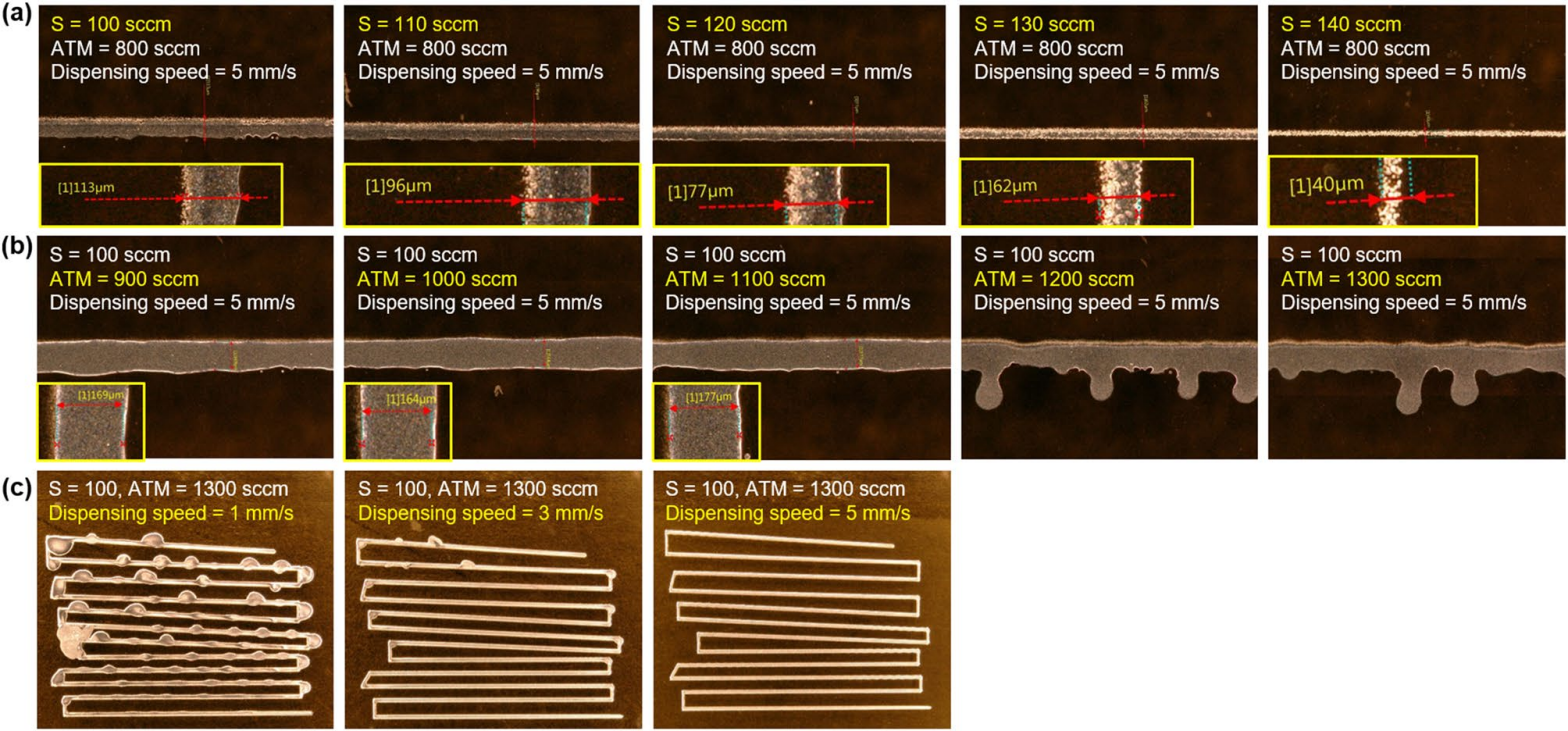
Verification of the tendency of operating conditions in AJ printing using other silver ink on a flexible substrate: Influence of (**a**) sheath flow rate, (**b**) atomizer flow rate, and (**c**) dispensing speed.

Figure 6 presents the results of verifying the feasibility by printing a different silver ink on a polyimide (PI) film, based on the observed tendency in AJ printing process parameters. The printing experiment utilized a nanoparticle silver ink (PRELECT® TPS 50G2, CLARIANT Co., Ltd.), which had an ink viscosity range of 50 ± 20 cPs, five times higher than that of the ink used in most of our experiments. Although higher viscosity alters the properties of the ink and differs in absolute values from the experimented parameters, the verification was focused primarily on the tendency. In Fig. 6a,b, the observed trend aligns with the decrease in printed line width as sheath flow rate increases and the increase in line width as atomizer flow rate increases. Due to a low evaporation rate and viscosity of ink, aerosol formation generates a stream at relatively high pressures^28,46^. The viscosity of ink is one of the factors that define the Stokes number, affecting the evaporation rate. This is associated with the drying and pressure of aerosols passing through the system due to carrier gas, ultimately determining the critical power required for atomization^28^. Deviations in process parameters largely depend on the specific ink, underscoring the need for parameter optimization. Consequently, a stable line pattern can be achieved with sheath flow rates exceeding 100 sccm. In Fig. 6c, an increase in dispensing speed corresponds to an improvement in print quality, while excessive ink deposition results in noticeable clustering due to the viscosity of ink. This highlights the necessity of optimizing process conditions based on the ink viscosity and evaporation rate, proving the compatibility of the process on various substrates.

### Statistics analysis for optimization of pneumatic aerosol jet process

**Figure 7:**
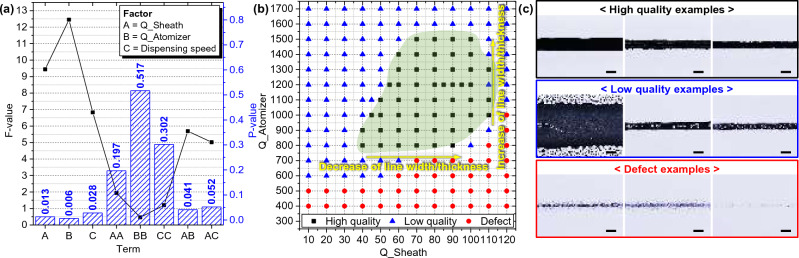
Response surface analysis and operability window of the process regime in terms of two operational parameters: (**a**) Analysis of each variance to identify the influence of parameters. (**b**) Classification of the printed line into three states as ‘high quality’, ‘low quality’, and ‘defect’ based on sheath and atomizer flow rate. (**c**) Optical microscopy image of the classified samples. (scale bar = 100 μm).

Figure 7a illustrates the results of response surface analysis along with F- and P-values. The response surface analysis is a valuable tool for determining the predominant factors contributing to response variability as a coefficient of factors in regression Eq. (4)^47,48^. Figure 7a shows the F-value and P-value, which can be used to determine the statistical significance of the association between the response and the factors^49,50^. The F-value is a testing statistic employed in regression analysis to gauge whether the coefficients of independent variables within the regression equation are equal to zero, signifying the degree of their impact on the dependent variable. An F-value closer to zero implies diminished impact, while an F-value greater than zero indicates a more pronounced departure from zero. Meanwhile, the P-value measures the likelihood of the F-value approximating zero. A smaller P-value indicates significant impact of independent variables, while a larger P-value suggests their diminished influence on the dependent variable. A conventional significance level of 0.05 is typically employed, signifying a 5% risk of concluding an association when none exists.

F- and P-values in Fig. 7a is allowing for the comparison of the relative magnitude and statistical significance of linear, quadratic, and two-way interaction effects involving three factors. The three defined factors are as follows: A, the sheath flow rate (Q_Sheath) with 8 levels (20, 40, 60, 80, 85, 90, 95, 100); B, the atomizer flow rate (Q_Atomizer) with 5 levels (400, 600, 800, 1000, 1200); and C, the dispensing speed with 3 levels (1, 5, 10). We incorporated quadratic forms to assess the square of each parameter (“AA”, “BB”, “CC”) and interaction terms (“AB”, “AC”) to better understand their effects on line width^47,48^. The reason for the absence of “BC” term is interaction effects are relatively small or within the margin of error, making them statistically insignificant. These factors collectively constitute the outcome of statistically analyzing 50 data points, resulting from the printing of 5 lines under each condition, with 10 measurement points per line. Upon reviewing the outcomes of the factor analysis, “B (Q_Atomizer)” exhibited the most significant impact on determining line width, followed by “A (Q_Sheath)” and “C (Dispensing speed).” This is because atomizer flow rate is that the atomizer is responsible for regulating the initial quantity of aerosol generation, thereby directly affecting the printed line width in terms of aerodynamics^34^. The remaining factors, sheath flow rate and dispensing speed control the density of silver within the generated aerosol and the processing speed, causing their impact relatively indirect^51^. The interaction terms “AB” and “AC” were found to be statistically insignificant. Additionally, the square terms, such as “AA” and “BB,” exhibited less influence on line width, and thus were not included in developing the regression Eq. (4)^47,48^. This analysis suggests that each parameter, including “A”, “B”, and “C” act independently as a linear parameter without significantly influencing other parameters when determining line width. In essence, this finding underscores that the three process parameters are not mutually dependent on each other and have distinct, individual effects on the outcome. To predict line width of aerosol printed line based on the response surface analysis, the empirical equations were derived in the form of Eq. (3) by considering the main effects and interactions of the analyzed process variables^52,53^.

3 $${\text{Line}}\;{\text{width }}\left( {A,B,C} \right) = a_{1} + a_{2} {\text{A}} + a_{3} {\text{B}} + a_{4} {\text{C}} + a_{5} AB + a_{6} AC + a_{7} BC + a_{8} A^{2} + a_{9} B^{2} + a_{10} C^{2}$$

Polynomial regression analysis was conducted to derive mathematical modeling that can predict the line width through the main effect and interaction analysis of the process variables that affect the line width analyzed previously. The significance analysis of each coefficient revealed that the significance of the coefficient values for the two-factor terms A, B, and C was relatively low. These terms were therefore pooled as error terms, and only the significant effects were included in the model, leading to the derivation of the analysis results as shown in Eq. (4). Based on this analysis, a regression equation including each factor for estimating line width printed by aerosol jetting was formulated:

4 $${\text{Line}}\;{\text{width }}\left( {\upmu {\text{m}}} \right) = 97.8 - 1.287{\text{A}} + 0.1033{\text{B}} - 5.43{\text{C}}$$

The analysis of variance showed a P-value of ≤ 0.05, and the adjusted coefficient of determination was 76.4%, indicating that the regression model is deemed appropriate. Through response surface design, the optimal line width and resistivity for each condition were analyzed. This analysis allows us to improve the experimental results and achieve more optimal control over the line width in aerosol jet printing.

Figure 7b presents the results of designing the operability window for aerosol jet printing based on the previous statistical analyses^54,55^. The primary influencing factors were chosen for the operability window in accordance with the principle of aerodynamics. The atomizer and sheath flow rate are associated with both quantity and quality of aerosol, a conclusion supported by the statistical analysis presented in Fig. 7a. Meanwhile, the dispensing speed is a parameter used for straightforward control of the line width, as dispensing speed and line width are inversely proportional. This is the reason for excluding it as a parameter in the operability window. Considering the necessity for the electrode application, the samples were evaluated across three levels with respect to resistivity. These levels include: (1) ‘high quality,’ characterized by successful resistivity measurement and intact form; (2) ‘low quality,’ characterized by successful resistivity measurement but flawed form; and (3) ‘defect,’ involving resistivity measurement failure. The printing results for each level can be referenced from Fig. 7c. Figure 7a revealed that the significant factors influencing line width are the atomizer and sheath flow rates. Thus, utilizing these two process parameters, the operability window was designed. The ink used in the experiment can be configured by referring to the graph within the range of atomizer flow rates between 800 and 1500 sccm, thereby determining the suitable sheath flow rate. Line width can be estimated using the regression Eq. (4). In conclusion, this experiment successfully established a system capable consistently achieving the desired line width for printed wiring using the operability window, providing a convenient and practical approach.

### Process feasibility verification for industrial applications

**Figure 8:**
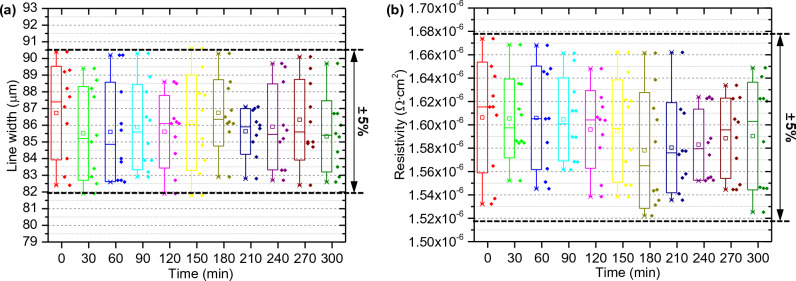
Process stability assessment of the pneumatic aerosol jet process leading to yield rate: Evaluation criteria include (**a**) Line width and (**b**) resistivity, printed at 90 sccm of sheath flow rate and 800 sccm of atomizer flow.

Figure 8 presents the results of verifying the process stability to demonstrate the feasibility for industrialization of the aerosol jet process. Since factors that affect production yield largely involve electrode formation, process stability assessment is essential for electrode formation processes. The samples used for evaluation were printed with sheath flow rate of 90 sccm, atomizer flow rate of 800 sccm, and dispensing speed of 5 mm/s. Process stability was defined by evaluating the changes in line width and resistivity values over process time and cycle. Thus, the approach involved printing line patterns every 30 min, measuring the line width and resistivity for 10 patterns each time. Examining the line width results over time in Fig. 8a, it can be observed that the variations for each time point remain within ± 4% based on the measurements of 10 patterns at each time. Furthermore, even as the printing time increases, there is minimal difference in the line width of the printed patterns, staying within ± 5% difference. The resistivity results in Fig. 8b also show a similar tendency, where the measurements for each time period, comprising 10 samples, change within ± 3% variation. Even with continuous printing over 5 hours, there is no significant difference exceeding ± 4% in the resistivity. Ultimately, compared to commercial inkjet and dispensing processes, aerosol jet printing breaks down any ink into small droplets, reducing the risk of nozzle clogging, thereby ensuring high process stability and the ability to form precise patterns. This highlights the potential of aerosol jet processes as a highly stable method that can be applied in practical industries.

## Conclusion

This study executed a line pattern process by controlling three parameters: sheath flow rate, atomizer flow rate, and dispensing speed, using a pneumatic aerosol jet printing machine. A systematic investigation of aerosol jet printing process parameters reveals conditions for generating high-quality conductive lines. The study analyzed the influence of adjustable parameters on the aerosol jetting process, based on the quality of printed line patterns and its resistivity. Examination of the printed line shapes indicates that sheath gas and atomizer flow rate significantly influence line printing quality, while dispensing speed controls the ink quantity per unit area, thereby governing the line width. This tendency remains consistent even when employing different viscosity silver inks and substrates. Additionally, the substrate temperature was verified as an additional parameter to control the line width and droplet diameter, which depends on the evaporation rate of ink and the spread time of printed ink. The statistical factor analysis was conducted to analyze the effects of each factor on line width, employing F-values and P-values, and deriving regression equations to estimate line width. Furthermore, operability windows were designed for the two main factors: sheath gas and atomizer flow rate. Building upon the regression equations and operability windows, a system was well established to print high-quality line patterns that meet the desired width conditions. Finally, the stability of the aerosol jet process, which has a substantial impact on production yield, was assessed by measuring changes in line width and resistance with respect to process cycles and time. The factor analysis of the proposed aerosol jet process in this study establishes a database that can be conveniently utilized in the industry, using an optimization approach based on ink viscosity and evaporation rate. Ultimately, pneumatic aerosol jet printing is a highly valuable research domain for the electrode formation process of flexible, rollable, and stretchable components, an area currently seeing significant research interest.

## Methods

### Printing process of pneumatic aerosol jet

All printing experiments were carried out with an ink containing soluble silver cluster & complex type (TEC-IJ-060, InkTec Co., Ltd.). This ink has a viscosity of 10 cPs and it is compatible with the pneumatic atomizer of the aerosol jet printer, which has a recommended ink viscosity range from 1 to 1000 cPs. The ink contains 12% by weight of the silver cluster in a solvent. For each printing run, an atomizer jar was loaded with 8 mL of the ink.

Silver line patterns were printed in a single pass using a commercial Aerosol Jet Printer (AEROSOL JET HD, OPTOMEC Inc.). Dry N_2_ (HP grade, 99.999%) was used as the carrier and sheath gas. Experiments were conducted with carrier gas and sheath gas flow rates ranging from 400 to 1700 standard cubic centimeters per minute (sccm) and from 10 to 120 sccm, respectively. The flow rate of the virtual impactor exhaust, divert, and boost was set as 0.185 psi, 100 sccm, and 100 sccm. The nozzle was used with a diameter of 300 μm. The atomizer current was kept constant at 0.28 A and the printer stage heater was not used due to the clogging issue. Stage speeds varied from 1 to 10 mm/s. Printing was done both on silicon wafer and polyimide (PI) film. The ink showed good wetting on substrates and hence no surface pretreatment was required. Unless otherwise noted, the results show data of printing on silicon wafers. The printed lines were sintered in a hotplate at 150°C for 5 minutes until the color of the ink changed from transparent to gray.

### Measurement and characterization

The printed line patterns were observed using optical microscopy (VHX-5000, KEYENCE Co., Ltd.) to measure the line width and tendency according to the operating conditions. The magnification was mostly used as 500×, and 1000×. The thickness and cross-sectional area of printed line patterns were characterized by confocal microscopy (VK-X200K, KEYENCE Co., Ltd.) on silicon wafers. The resistance of the line pattern printed length of 1 cm was measured with a precise resolution source meter (2634B, KEITHLEY INSTRUMENTS, Inc.) inside of the probe station (EPS200RF, Cascade Microtech Inc.). The source, and measure function was set to voltage and current, separately. The source mode was DC bias, and the level of voltage was used as 1 V. Furthermore, the resistivity was calculated from the equation: ρ is electrical resistivity, R is measured line resistance, A is the cross-sectional area of the printed pattern, and L is printed line length.

$$\uprho = \frac{R \cdot A}{L}$$

## Data Availability

The datasets used and/or analyzed during the current study available from the corresponding author on reasonable request.
